# A Postsurgical Prognostic Nomogram for Locally Advanced Rectosigmoid Cancer to Assist in Patient Selection for Adjuvant Chemotherapy

**DOI:** 10.3389/fonc.2021.772482

**Published:** 2021-12-24

**Authors:** Chao Zhang, Shutao Zhao, Xudong Wang

**Affiliations:** Department of Gastrointestinal Nutrition and Hernia Surgery, The Second Hospital of Jilin University, Changchun, China

**Keywords:** stage II/III rectosigmoid junction cancer, nomogram, prognosis, adjuvant chemotherapy, surgery

## Abstract

**Background:**

The perioperative treatment model for locally advanced rectosigmoid junction cancer (LARSC) has not been finalized; whether this model should refer to the treatment model for rectal cancer remains controversial.

**Methods:**

We screened 10,188 patients with stage II/III rectosigmoid junction adenocarcinoma who underwent surgery between 2004 and 2016 from the National Cancer Institute Surveillance, Epidemiology, and End Results database. Among them, 4,960 did not receive adjuvant chemotherapy, while 5,228 did receive adjuvant chemotherapy. Propensity score matching was used to balance the two groups for confounding factors, and the Kaplan-Meier method and log-rank test were used for survival analysis. Cox proportional hazards regression analysis was used to identify independent prognostic factors and build a predictive nomogram of survival for LARSC. X-tile software was used to divide the patients into three groups (low, medium, and high) according to their risk scores. 726 patients in our hospital were included for external validation.

**Results:**

LARSC patients did not show a benefit from neoadjuvant radiotherapy (P>0.05). After further excluding patients who received neoadjuvant radiotherapy, multivariate analysis found that age, grade, tumor size, T stage, and log odds of positive lymph nodes were independent prognostic factors for patients without adjuvant chemotherapy and were included in the nomogram. The C-index of the model was 0.690 (95% confidence interval: 0.668–0.712). We divided the patients into low, moderate, and high risk subgroups based on prediction scores of the nomogram. We found that adjuvant chemotherapy did not improve the prognosis of low risk patients, while moderate and high risk patients benefited from adjuvant therapy. External validation data found that moderate, and high risk patients also benefited from AT.

**Conclusion:**

Direct surgery plus adjuvant chemotherapy may be the best perioperative treatment for LARSC. Moreover, adjuvant chemotherapy is only recommended for moderate and high risk patients as it did not benefit low risk patients.

## Background

Rectal cancer (RC) is the eighth most common cancer and the ninth leading cause of cancer-related deaths worldwide (1). The rectosigmoid junction is located in the sigmoid colon, but its surgical approach refers to the rectum, so it is now considered a separate site (2). Locally advanced RC usually refers to clinical stage II/III middle and low RC. The traditional treatment method for such patients is the “sandwich mode” (radiotherapy-surgery-chemotherapy), which reduces the local recurrence rate from 35% to 10%, while the distant metastasis rate remains approximately 25%. To reduce the distant metastasis rate of RC, its treatment mode has been changed to total neoadjuvant therapy (TNT) (3–7). Adjuvant chemotherapy (AT) is now only recommended if there are high risk factors for stage II colon cancer (8).

Neoadjuvant radiotherapy is usually not recommended for locally advanced colon cancer, as surgery plus AT is recommended; in contrast, preoperative radiotherapy is recommended for RC (9, 10). However, the perioperative treatment plan for locally advanced rectosigmoid junction cancer (LARSC) is controversial, especially regarding whether radiotherapy should be given prior to surgery or whether chemotherapy should be given after surgery. The current guidelines do not contain guidance in this regard. Therefore, the focus of this study was to determine whether LARSC patients benefited from neoadjuvant radiotherapy, especially whether the mode of surgery plus adjuvant therapy was suitable for all patients or whether there was overtreatment in certain patient subgroups that received adjuvant therapy.

To this end, the Surveillance, Epidemiology, and End Results (SEER) database was used to analyze whether locally advanced RC patients benefited from neoadjuvant radiotherapy. The patients were further divided into three subgroups of low, moderate, and high risk according to nomogram scores, and the patients who benefited from AT were selected and further verified using external data from our center.

## Methods

### Patient Cohorts

SEER*Stat (version 8.3.9) software was used to identify 15,455 patients diagnosed with p stage II/III rectosigmoid junction cancer between 2004 and 2016. The inclusion criteria were as follows (1): patients with pathologically confirmed rectosigmoid junction cancer (ICD-O-3: C19.9) (2); adenocarcinoma (3); primary rectosigmoid junction cancer (4); patients underwent definite surgery (anti-tumor resection with abdominoperineal research); and (5) did not receive neoadjuvant radiotherapy. According to whether the patients had received AT, they were divided into the non-adjuvant therapy group (4,960) and AT group (5,228). The included variables were age, sex, grade, tumor size, T stage, log odds of positive lymph nodes (LODDS), chemotherapy information, and survival time. If any of the above variables were unknown, the patient was excluded.

The external validation data included 726 patients with stage II/III rectosigmoid junction cancer diagnosed by pathology at our hospital between 2013 and 2016. The inclusion criteria and analyzed variables were the same as for the SEER cohort.

### Statistical Analysis

Before excluding patients who received neoadjuvant radiotherapy, the Kaplan-Meier method and log-rank test were used to analyze whether LARSC patients benefited from neoadjuvant radiotherapy. After excluding neoadjuvant radiotherapy patients, the best cut-off values for LODDS were obtained using X-tile software. The chi-square test was used to analyze the relationship between the two groups (without AT and with AT) and clinicopathological factors. To balance confounding biases of the included cases, significant clinicopathological factors from the chi-square test were included for propensity score matching (PSM), and nearest neighbor matching was performed at a ratio of 1:1 in the non-AT and AT groups (11). Then Kaplan-Meier curves and the log-rank test were used to compare survival between the two groups.

The predictive model was established as follows (1): univariate and multivariate Cox analyses were used to analyze correlations between variables and overall survival (OS) in the non-AT group (2); using results from the Cox multivariate analysis, variables with P<0.05 were selected to establish a nomogram (3); the efficacy of the prediction model was tested using discrimination, which was measured by the concordance index (C-index) (12) (4); the clinical efficacy of the C-index evaluation model of the nomogram and T stage was compared (5); a calibration curve was generated that showed the consistency between the predicted survival rate and the actual survival rate (6); decision curve analysis (DCA) was used to evaluate the clinical net benefit and compare with T stage (13); and (7) the patients were divided into three subgroups (high, moderate, and low risk) according to the nomogram risk scores using X-tile software, and then the benefits from AT in the three subgroups were evaluated (14). SPSS (version 24.0; IBM, Armonk, NY, USA) and R language (version 4.0.0) were used for all statistical analyses. P<0.05 was considered statistically significant.

## Results

### Patient Demographics

**Figure 1:**
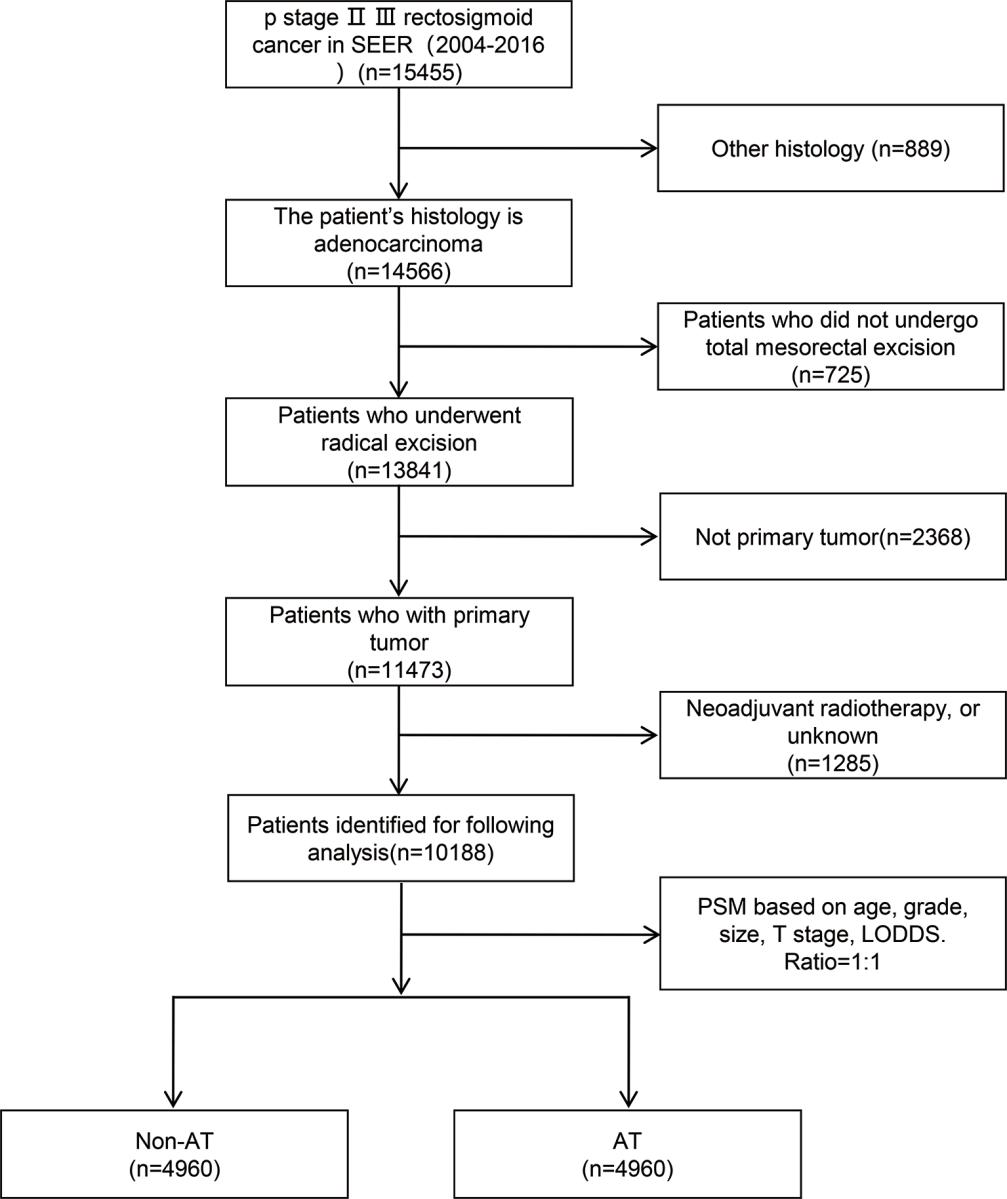
Flowchart of the selection process of included patients.

**Figure 2:**
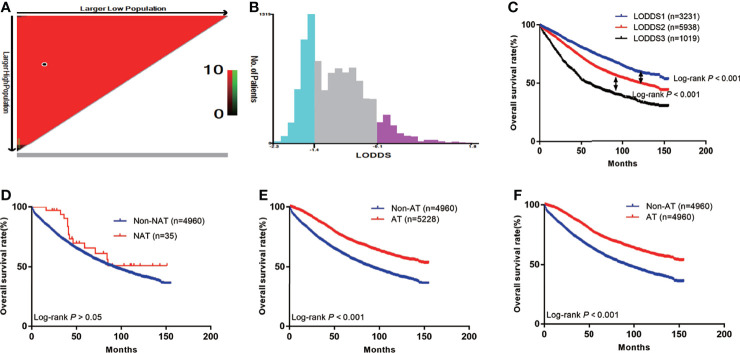
The Kaplan-Meier curves of OS for patients in our study. **(A)** The optimal cut-off value; **(B)** Numbers of patients in three subgroups; **(C)** OS in different subgroups of all patients; **(D)** OS in non-NAT and NAT group; **(E)** OS in non-AT and AT group before PSM; **(F)** OS in non-AT and AT group after PSM.

In total, 4,995 patients did not receive AT, among whom 35 received neoadjuvant radiotherapy and 4,960 did not (**Figure 1**). The survival curve showed that neoadjuvant radiotherapy did not improve patient survival [hazard ratio (HR)=0.73; 95% confidence interval (CI): 0.46–1.17; P>0.05] (**Figure 2D**), since relatively few patients receive neoadjuvant radiotherapy, our results may need further validation. First, patients who received neoadjuvant radiotherapy were further excluded, leaving 10,188 locally advanced patients with radical surgery, including 4,960 without AT and 5,228 with AT. The median survival time of patients was 53 months (range: 0–155 months), and there were 3,843 deaths (37.7%). We divided the LODDS into three groups by cut-off values (**Figures 2A–C**). The chi-square test showed that AT was significantly correlated with age, grade, tumor size, T stage, and LODDS (all P<0.05). After including the variables significantly related to AT for PSM, the final number of patients was 9,920, with 50% (*n*=4,960) in the groups with and without AT. The median survival time of this patient cohort remained 53 months (range: 0–155 months), and there were 3,757 deaths (37.9%) (**Table 1**).

**Table 1:** Characteristics of patients.

Variable	Unmatched Cohort			P value	Matched Cohort			P value
	Total [n (%)]	Non-AT [n (%)]	AT [n (%)]		Total [n (%)]	Non-AT [n (%)]	AT [n (%)]	
Age	10188	4960	5228	<0.001	9920	4960	4960	<0.001
<65	5197	1756 (35.4)	3441 (65.8)		5197	1756 (35.4)	3441 (69.4)	
≥65	4991	3204 (64.6)	1787 (34.2)		4723	3204 (64.6)	1519 (30.6)	
Sex				0.8344				0.783
Male	5671	2700 (54.4)	2971 (56.8)		5525	2700 (54.4)	2825 (57.0)	
Female	4517	2160 (45.6)	2357 (43.2)		4395	2160 (45.6)	2235 (43.0)	
Grade				<0.001				<0.001
Well/moderately	8488	4243 (85.5)	4245 (81.2)		8220	4243 (85.5)	3977 (80.2)	
Poorly/undifferentiated	1574	651 (13.2)	923 (17.7)		1574	651 (13.1)	923 (18.6)	
Unknown	126	66 (1.3)	60 (1.1)		126	66 (1.4)	60 (1.2)	
Size (cm)				<0.001				<0.001
<3	1376	605 (12.2)	771 (14.7)		1376	605 (12.2)	771 (15.5)	
≥3	8812	4355 (87.8)	4457 (85.3)		8544	4355 (87.8)	4189 (84.5)	
T stage				<0.001				<0.001
T1	234	86 (1.7)	148 (2.8)		234	86 (1.7)	148 (3.0)	
T2	748	242 (4.9)	506 (9.7)		739	242 (4.9)	497 (10.2)	
T3	7772	4066 (82.0)	3706 (70.9)		7574	4066 (82.0)	3508 (70.7)	
T4	1434	566 (11.4)	868 (16.6)		1373	566 (11.4)	807 (16.1)	
LODDS				<0.001				<0.001
LODDS1≤-1.4	3231	2139 (43.1)	1092 (20.9)		2963	2139 (43.1)	824 (16.6)	
-1.4<LODDS2≤-0.1	5938	2488 (50.2)	3450 (66.0)		5938	2488 (50.2)	3450 (69.6)	
-0.1<LODDS3 ≤ 1.8	1019	333 (6.7)	686 (13.1)		1019	333 (6.7)	686 (13.8)	

AT, adjuvant chemotherapy; LODDS, log odds of positive lymph nodes.

The prognoses of patients with AT before PSM was higher than that of the non-AT group (5-year OS: 75.2% vs. 60.7%; P<0.001) (**Figure 2E**). After PSM, the prognosis of patients with AT remained higher than that of the non-AT group (5-year OS: 75.2% vs. 60.7%; P<0.001) (**Figure 2F**).

### Nomogram Construction

**Figure 3:**
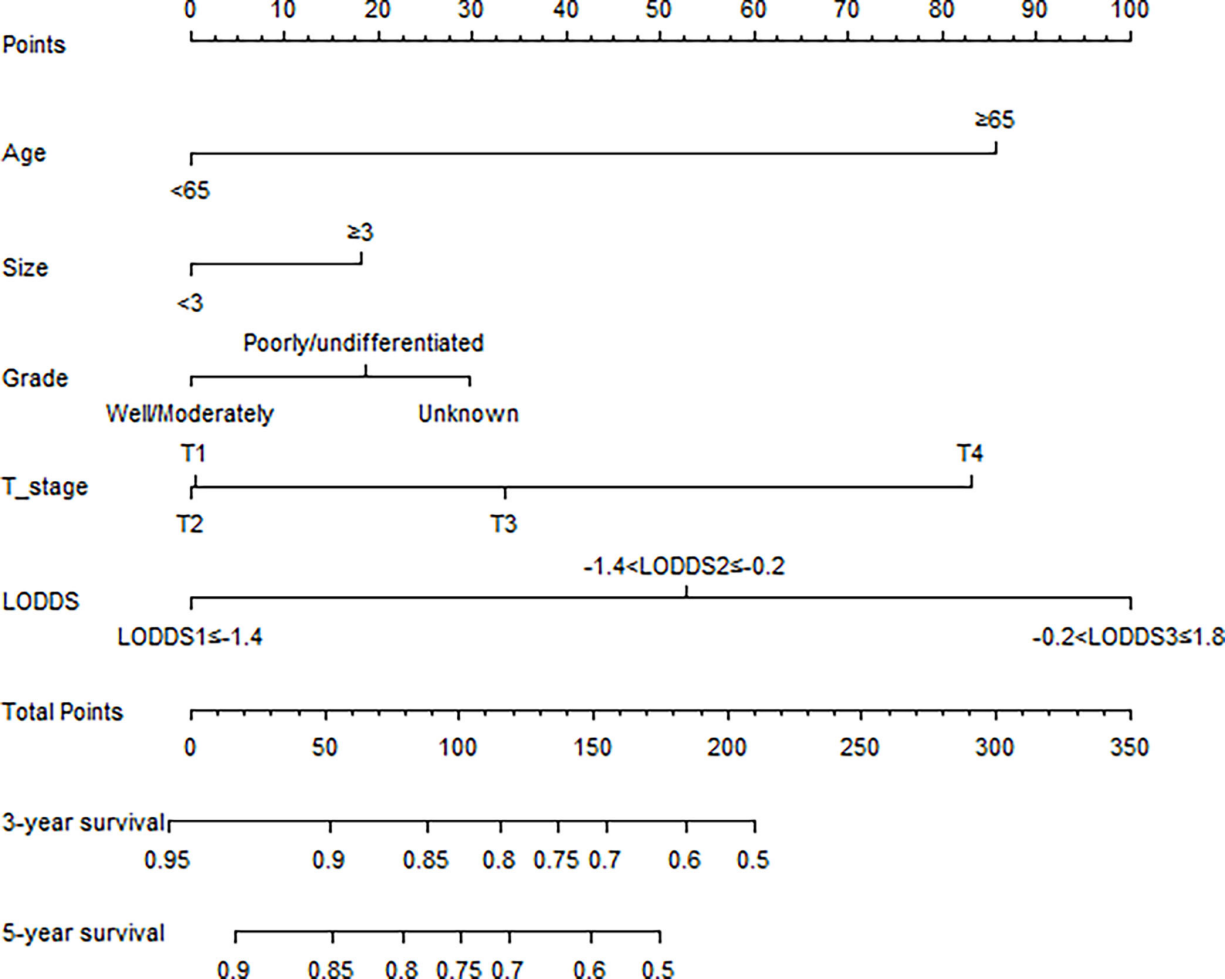
Oncologic nomogram for rectosigmoid cancer patients.

To exclude the effect of AT on patient survival, patients without AT were included in the Cox proportional hazard model. Univariate analysis showed that age, grade, tumor size, T stage, and LODDS were associated with OS (all P<0.05). Multivariate analysis showed that age, grade, tumor size, T stage, and LODDS were all independent prognostic factors (P<0.05) (**Table 2**). On this basis, a nomogram was constructed to predict 3- and 5-year OS rates, and a risk score for each prognostic factor was calculated (**Figure 3**).

**Table 2:** The univariate and multivariate analyses of factors associated with overall survival.

Variable	Univariate Cox regression		Multivariate Cox regression	
	HR (95% CI)	P-value	HR (95% CI)	P-value
Age				
<65	1			
≥65	2.929 (2.640-3.248)	<0.001	2.761 (2.489-3.063)	<0.001
Sex				
Male	1			
Female	0.992 (0.915-1.077)	0.855		
Grade				
Well/Moderately	1			
Poorly/undifferentiated	1.455 (1.302-1.625)	<0.001	1.242 (1.111-1.390)	<0.001
Unknown	1.103 (0.778-1.564)	0.582	1.416 (0.997-2.012)	0.052
Size (cm)				
<3	1			
≥3	1.267 (1.112-1.444)	<0.001	1.238 (1.079-1.421)	0.002
T stage				
T1	1			
T2	1.133 (0.767-1.676)	0.530	0.996 (0.669-1.481)	0.983
T3	1.314 (0.931-1.854)	0.120	1.478 (1.033-2.115)	0.033
T4	2.652 (1.856-3.788)	<0.001	2.651 (1.828-3.844)	<0.001
LODDS				
LODDS1≤-1.4	1			
-1.4<LODDS2≤-0.2	1.937 (1.767-2.123)	<0.001	1.870 (1.703-2.053)	<0.001
-0.2<LODDS3 ≤ 1.8	3.543 (3.062-4.099)	<0.001	3.255 (2.801-3.782)	<0.001

LODDS, log odds of positive lymph nodes.

### Testing the Effectiveness of the Prediction Model

**Figure 4:**
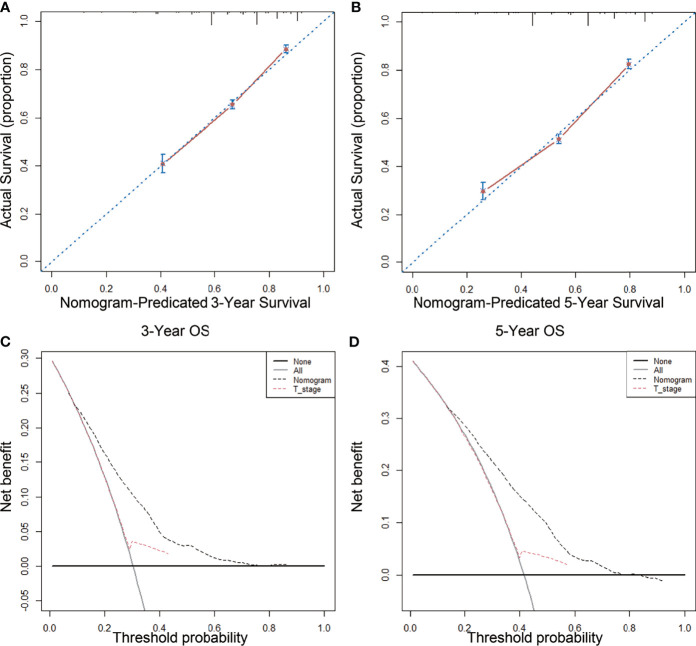
Calibration curves and decision curve for OS prediction: **(A)** 3-year OS calibration curve in our cohort; **(B)** 5-year OS calibration curve in our cohort; **(C)** Nomogram were compared to the T stage in terms of 3-year OS in our decision curve analysis; **(D)** Nomogram were compared to T stage in terms of 5-year OS in our decision curve analysis.

The nomogram model that incorporated the above five risk factors had a C-index of 0.690 (95% CI: 0.668–0.712) for prognosis, which is significantly higher than the C-index for prognostic judgment according to T stage [0.550 (95% CI: 0.532–0.568)]. The nomogram calibration curves for 3- and 5-year OS showed that the predicted survival probability was consistent with the actual survival probability (**Figures 4A, B**). DCA showed that the prognostic nomogram model had higher net yield for different decision thresholds than the prediction line of the T staging system (**Figures 4C, D**).

### Risk Stratification System for the Study Cohort

**Figure 5:**
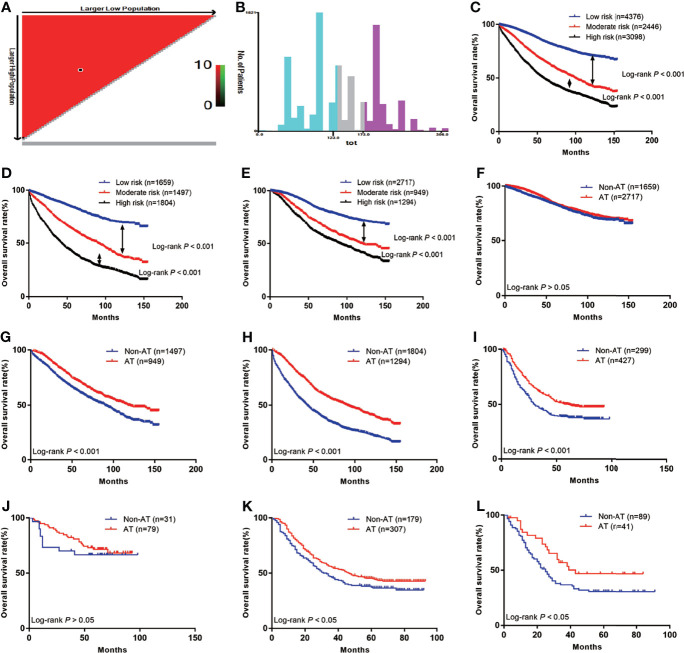
The Kaplan-Meier curves of OS for subgroups in our study: **(A)** The optimal cut-off value; **(B)** Numbers of patients in low, moderate and high risk subgroups; **(C)** OS in different subgroups of all patients; **(D)** OS in different subgroups of non-AT group; **(E)** OS in different subgroups of AT group; **(F)** OS for patients with or without AT in low risk group; **(G)** OS for patients with or without AT in moderate risk group; **(H)** OS for patients with or without AT in high risk group; **(I)** OS for patients with or without AT in external validation group; **(J)** OS for external validation patients with or without AT in low risk group; **(K)** OS for external validation patients with or without AT in moderate risk group; **(L)** OS for external validation patients with or without AT in high risk group.

First, we calculated the total risk score for each patient in the two groups using the nomogram (**Table 3**), and then divided the patients into three subgroups according to two cut-off values obtained using X-tile software: the low risk group (score: ≤123, *n*=4376), the moderate risk group (score: 123–173, *n*=2446), and the high risk group (score: 174–306, *n*=3098) (**Figures 5A, B**), The 5-year OS rates of the low, moderate, and high risk groups were 83.8%, 65.1%, and 48.6%, respectively, which represented statistically significant differences (P<0.001) (**Figure 5C**). In non-AT group, the 5-year OS rates of the low, moderate, and high risk groups were 83.4%, 61.2%, and 39.6%, respectively, which represented statistically significant differences (P<0.001) (**Figure 5D**). In AT group, the 5-year OS rates of the low, moderate, and high risk groups were 84.0%, 71.0%, and 60.4%, respectively, which represented statistically significant differences (P<0.001) (**Figure 5E**) (**Table 4**).

**Table 3:** Point assignment of each component and prognostic score for rectosigmoid cancer.

Group	Score	Estimated 3-y OS (%)	Estimated 5-y OS (%)
Age			
<65	0		
≥65	86		
Grade			
Well/moderately	0		
Poorly/undifferentiated	19		
Unknown	30		
Size (cm)			
<3	0		
≥3	18		
T stage			
T1	0		
T2	0		
T3	33		
T4	83		
LODDS			
LODDS1≤-1.4	0		
-1.4<LODDS2≤-0.1	53		
-0.1<LODDS3 ≤ 1.8	100		
Total score			
	-8	95	
	52	90	
	89	85	
	115	80	
	136	75	
	155	70	
	185	60	
	210	50	
	16		90
	53		85
	79		80
	101		75
	119		70
	149		60
	175		50

LODDS, log odds of positive lymph nodes.

**Table 4:** Risk stratification in non-AT and AT group.

Survival status	Non-AT Group			P value	AT Group			P value
	Low Risk [n (%)]	Moderate Risk [n (%)]	High Risk [n (%)]		Low Risk [n (%)]	Moderate Risk [n (%)]	High Risk [n (%)]	
Live	1301 (78.4)	794 (53.0)	547 (30.3)	<0.001	2206 (81.2)	614 (64.7)	701 (54.2)	<0.001
Death	358 (21.6)	703 (47.0)	1257 (69.7)		511 (18.8)	335 (35.3)	593 (45.8)	

AT, adjuvant chemotherapy.

### Evaluating the Efficiency of Adjuvant Therapy for Patients in Different Groups

We further investigated whether the low, moderate, and/or high risk patients benefited from AT. The results showed that patients in the low risk group did not benefit from AT (HR=0.87, 95% CI: 0.76–1.00; P>0.05) (**Figure 5F**), while the moderate and high risk groups benefited from AT (HR=0.70, 95% CI: 0.62–0.79; P<0.001 and HR=0.53, 95% CI: 0.48–0.58; P<0.001, respectively) (**Figures 5G, H**).

### Evaluating the Efficiency of Adjuvant Therapy for Patients in the External Validation Group

In total, 726 LARSC patients who underwent surgery at our center were included. The specific pathological conditions of this cohort are shown in **Table S1**, including 299 patients who were not treated with AT and 427 patients were treated with AT. The median survival was 41 months (range: 0–93 months), and there were 400 deaths (55.1%). The prognoses of patients with AT was improved compared with those without AT (5-year survival: 50.2% vs. 38.4%; P<0.001) (**Figure 5I**). According to the above scoring system, the external validation cohort were also divided into low, moderate, and high risk groups, and these results confirmed that the low risk group did not benefit from AT (HR=0.76, 95% CI: 0.34–1.67; P>0.05) (**Figure 5J**). In contrast, the moderate and high risk groups benefited from AT (HR=0.78, 95% CI: 0.60–1.00; P=0.01 and HR=0.59, 95% CI: 0.37–0.94; P=0.04, respectively) (**Figures 5K, L**).

## Discussion

Because the blood supply to rectosigmoid junction cancer is the same as the blood vessels of upper RC, rectosigmoid junction cancer is most often defined as RC (15). Compared with locally advanced RC, the latest guidelines recommend that low risk LARSC patients receive TNT or traditional “sandwich mode” therapy (radiotherapy-surgery-chemotherapy), while TNT is recommended for high risk patients. TNT refers to the transfer of all postoperative AT to preoperative AT on the basis of preoperative concurrent chemoradiotherapy or short-term radiotherapy, which constitutes neoadjuvant chemoradiotherapy. Finally, total mesorectal excision is performed, and AT is ceased (16). The advantage of preoperative radiotherapy over non-preoperative radiotherapy is that it reduces the local recurrence rate (7.1% vs. 10.1%; p=0.048) but does not reduce the distant recurrence rate (29.8% vs. 29.6%; P=0.9) or improve long-term survival (17). Breugom et al. included four large randomized controlled studies and found that after neoadjuvant chemoradiation for advanced RC, postoperative AT did not improve OS (HR=0.97, 95% CI: 0.81–1.17; P=0.775) or disease-free survival (HR=0.91, 95% CI: 0.77–1.07; P=0.230) (18–22). Therefore, it remains unclear whether preoperative neoadjuvant radiotherapy benefits LARSC patients and whether postoperative chemotherapy is suitable for all patients. The purpose of this study was to determine a suitable perioperative treatment plan for LARSC patients.

Our study found no benefit from preoperative radiotherapy (HR=0.73, 95% CI: 0.46–1.17; P>0.05). Neoadjuvant radiotherapy can significantly reduce tumor volume and improve the resection rate and radical cure rate. It can also reduce tumor invasion to the surrounding tissue, which also reduces the number of positive surrounding lymph nodes. Thus, preoperative radiotherapy can decrease clinical stage. Tumor fibrosis results in reduced cell viability, making the possibility of implantation very low, even if cells are shed during surgery. The long-term advantage of preoperative radiotherapy is to reduce the local recurrence rate (23–25). Deng et al. (26) found no statistical differences in OS between direct surgery and neoadjuvant radiotherapy in low-risk patients (HR=1.486, 95% CI: 0.716–3.087; P=0.287), in the local recurrence rate (HR=1.018, 95% CI: 0.205–5.046; P=0.983), or in the distant recurrence rate (HR=1.675, 95% CI: 0.812–3.455; P=0.163). In high risk patients, direct surgery had no effect on local or distant recurrence rates compared with neoadjuvant radiotherapy (P>0.05). One possible reason was that the sensitivity of adenocarcinoma to radiotherapy was lower than that of squamous cell carcinoma. Neoadjuvant radiotherapy is relatively suitable for RC in the middle and low position, i.e., below the peritoneal reflex. Therefore, neoadjuvant radiotherapy for LARSC may constitute overtreatment.

The latest meta-analysis included 29 articles and found that AT improved the OS of stage II colorectal cancer patients (HR=0.61, 95% CI: 0.54–0.68; P<0.001), but only in high risk patients (T4 patients, poorly differentiated or undifferentiated cancers, lymphovascular or perineural invasion, intestinal obstruction or perforation, <12 retrieved lymph nodes, and positive margins) (27). AT is recommended for patients with stage II colon cancer only if there are high risk factors, and this meta-analysis contained colon cancer and RC, so not all LARSC patients will be suitable for AT. Moreover, AT is only recommended for high risk patients. Therefore, we studied risk factors associated with postoperative recurrence in LARSC, which could significantly contribute to guiding the AT regimens of LARSC patients.

Due to decreased immunity and of various other functions in elderly people, the incidence of tumors is relatively higher than in younger people. The elderly have insidious tumor onset, atypical clinical symptoms, and are often accompanied by other chronic diseases, resulting in relatively poor post-surgical prognoses (28). Our study found that the prognosis of elderly LARSC patients was poor (HR=2.761, 95% CI: 2.489–3.063; P<0.001). Previous studies have found that aspirin may improve the prognosis of elderly colorectal cancer patients (29). However, the latest study showed that aspirin did not reduce the incidence rate (HR=1.02, 95% CI: 0.81–1.30) or mortality rate (HR=1.77, 95% CI: 1.02–3.06) among elderly patients (30). Our study found that patients with poorly/undifferentiated tumors had poorer prognoses (HR=1.242, 95% CI: 1.111–1.390; P<0.001). The biological behaviors including invasion and metastasis of malignant tumors depends on the specific tumor histological type and degree of differentiation, which are key factors in judging tumor progression and prognosis. Patients with poorly/undifferentiated tumors have poor prognosis and were associated with peritoneal metastasis (31–33). Tumor size is one of the most important basic indicators of postoperative pathology and is related to the degree of tumor differentiation, T stage, N stage, and is considered an important factor for the prognosis of patients with solid tumors (34). Our study also found that tumors ≥3 cm were associated with poor prognosis (HR=1.238, 95% CI: 1.079–1.421; P=0.002).

The TNM staging system highlights the influence of depth of tumor invasion on treatment efficacy and prognosis, and further refines the T staging. Staging alone does not pay enough attention to the depth of tumor invasion. Regardless of T stage, as long as the N stage is N0, a tumor is stage I or II; additionally, regardless of T stage, as long as the N stage is not N0, a tumor is stage III. Gunderson et al. found that whether a tumor was N0, N1, or N2, with increasing T stage, the 5-year survival rate of patients gradually decreased. For example, the 5-year OS rates of patients with N0, T1, T2, T3, T4a and T4b were 96.6%, 92.1%, 78.7%, 69.2%, and 53.6%, respectively (35). Our study also found that T3 and T4 were poor prognostic factors. N staging is based on positive lymph nodes. With the increased study of negative lymph nodes, it has been found that N staging was dramatically affected by the number of lymph nodes retrieved, making this measure prone to stage migration. Patients with the same number of metastatic lymph nodes may have significantly different prognoses, thus affecting the accuracy of judging the prognosis of colorectal cancer patients. Therefore, LODDS may be a better prognostic indicator for colorectal cancer patients (36–38). Zhang et al. (39) confirmed that LODDS could be divided into three proportions. Within the three categories of increasing proportion, the 5-year OS of colorectal patients were 77.2%, 55.0%, and 26.7% respectively, which confirmed that LODDS was an independent prognostic factor. In our study, we used X-tile software to determine the best LODDS cut-off values, and found that with as the LODDS ratio increased, patient prognosis became worse. Lymph node metastasis is one of the primary modes of colorectal cancer metastasis that it is also an important cause of recurrence and mortality in patients following radical resection.

We established a nomogram by incorporating independent risk factors of the postoperative prognosis of LARSC patients. Our model had a C-index of 0.690 [95% CI: 0.668–0.712)] and could better predict 3- and 5-year OS after surgery. Our application of DCA further confirmed that the nomogram was superior to T stage at predicting OS.

AT is only recommended for high risk stage II colon cancer patients. Verhoeff et al. (40) found that only T4 patients (HR=0.43, 95% CI: 0.28–0.66) and those with two risk factors (HR=0.58, 95% CI: 0.43–0.80) benefited from chemotherapy; however, patients with poorly/undifferentiated tumors, emergency surgery for intestinal obstruction or perforation, and <10 lymph nodes did not benefit from AT. Both before and after PSM, our data showed that patients benefited from AT. We next evaluated whether all risk factors benefited from AT, if only certain combinations of multiple risk factors benefited, or if we could evaluate the risk factors of each patient independently, as individual patients could have multiple risk factors that affect prognosis or no risk factors at all. Therefore, we needed to accurately select the patients who would actually benefit from AT. To this end, we analyzed risk factors that most significantly affected the prognosis of LARSC by creating a nomogram, and then scored the prognostic impact of each high-risk factor. Finally, the patients were divided into three subgroups by obtained cut-off values. Moreover, in the surgery group and AT group, there were significant differences in survival among the subgroups of low, moderate, and high risk patients, indicating that our subgroups were reasonable and effective.

To study which patient subgroup benefited the most from chemotherapy, we investigated the 5-year OS rates, which revealed that 5-year survival of low risk patients who received AT was decreased compared with those who did not receive AT (84.0% vs. 83.4%; P>0.05); thus, AT is not recommended for low risk patients. The 5-year survival rate of moderate risk patients who received AT was higher than that of the non-AT group (71.0% vs. 61.2%; P<0.001). The 5-year survival rate of the high risk group was higher than that of the non-adjuvant group (60.6% vs. 39.6%; P<0.001); thus, AT is recommended for moderate and high risk patients.

Next, we analyzed data from our center and found that, according to the above risk scores and grouping criteria, the 5-year survival rate of low risk patients who received AT was slightly decreased compared with the non-AT group (71.5% vs. 66.5%; P>0.05). The 5-year OS rate of moderate risk patients who received AT was higher than that of the non-AT group (45.3% vs. 37.3%; P<0.05). The 5-year survival rate of patients in the high risk group who received adjuvant therapy was also higher than that of the non-adjuvant therapy group (46.9% vs. 30.7%; P<0.05). Thus, external validation using data from our center was consistent with the previous results. AT is not recommended for low-risk patients, while it is recommended for moderate and high risk patients. The relatively low survival rate among patients at our center is related to the relatively small number of patients and the relatively large proportion of stage III disease.

Finally, this study included some limitations. First, the number of patients who received neoadjuvant radiotherapy was lower than the number of patients who did not receive neoadjuvant radiotherapy, which may have impacted results on the role of neoadjuvant radiotherapy. Second, the AT regimens of patients were not uniform, and the degree of completion was not the same, which may have caused our results to be biased. Third, this was a retrospective study, which may lead to bias. However, our study provides a new perioperative plan for LARSC. Our model is of great significance for the individualized guidance of clinical AT, which highlights the importance of our article.

## Conclusion

In summary, our data showed that neoadjuvant radiotherapy is not recommended for LARSC. According to our risk score model, AT is only recommended for moderate and high risk LARSC patients.

## Data Availability Statement

The original contributions presented in the study are included in the article/**Supplementary Material**. Further inquiries can be directed to the corresponding author.

## Author Contributions

XW designed the research. CZ took part in designing the research. SZ collected the data, analyzed the date. CZ analyzed the date and wrote the manuscript. All authors contributed to the article and approved the submitted version.

## Funding

This work was supported by Department of Finance of Jilin Province (No 2020SCZT031, 2019SCZT023).

## Conflict of Interest

The authors declare that the research was conducted in the absence of any commercial or financial relationships that could be construed as a potential conflict of interest.

## Publisher’s Note

All claims expressed in this article are solely those of the authors and do not necessarily represent those of their affiliated organizations, or those of the publisher, the editors and the reviewers. Any product that may be evaluated in this article, or claim that may be made by its manufacturer, is not guaranteed or endorsed by the publisher.
