# Supplementary material for: A Postsurgical Prognostic Nomogram for Locally Advanced Rectosigmoid Cancer to Assist in Patient Selection for Adjuvant Chemotherapy
Source: Front Oncol. 2021 Dec 24;11:772482. doi: 10.3389/fonc.2021.772482 (PMC8739949; doi:10.3389/fonc.2021.772482)
Supplement: Supplementary file 1 [file Table_1.docx]

Table S1. Characteristics of patients

| Variable | Total [n（%）] | Non-AT[n（%）] | AT[n（%）] | P value |
| --- | --- | --- | --- | --- |
| Age | 726 | 299 | 427 | <0.001 |
| <65 | 476 | 136(45.5) | 340(79.6) |  |
| ≥65 | 250 | 163(54.5) | 87(20.4) |  |
| Sex |  |  |  | 0.541 |
| Male | 518 | 217(72.6) | 301(70.5) |  |
| Female | 208 | 82(27.4) | 126(29.5) |  |
| Grade |  |  |  | 0.309 |
| Well/moderately | 314 | 136(45.5) | 178(41.7) |  |
| Poorly/undifferentiated | 412 | 163(54.5) | 249(58.3) |  |
| Size (cm) |  |  |  | 0.640 |
| <3 | 70 | 27(9.0) | 43(10.1) |  |
| ≥3 | 656 | 272(91.0) | 384(89.9) |  |
| T stage |  |  |  | 0.353 |
| T1 | 29 | 14(4.7) | 15(3.5) |  |
| T2 | 76 | 25(8.4) | 51(11.9) |  |
| T3 | 171 | 75(25.1) | 96(22.5) |  |
| T4 | 450 | 185(61.8) | 265(62.1) |  |
| LODDS |  |  |  | 0.829 |
| LODDS1≤-1.4 | 146 | 57(19.1) | 89(20.8) |  |
| -1.4<LODDS2≤-0.1 | 137 | 58(19.4) | 79(18.5) |  |
| -0.1<LODDS3≤1.8 | 443 | 184(61.5) | 259(60.71) |  |

AT, adjuvant chemotherapy; LODDS, log odds of positive lymph nodes
